# Can ammonia scavenging treat MASLD? Evaluating the evidence for L‐ornithine L‐aspartate—A systematic review

**DOI:** 10.1111/eci.70185

**Published:** 2026-02-07

**Authors:** Abdulrahman Ismaiel, Vera Ciornolutchii, Stefan‐Lucian Popa, Dan L. Dumitrascu

**Affiliations:** ^1^ 2nd Department of Internal Medicine ‘Iuliu Hatieganu’ University of Medicine and Pharmacy Cluj‐Napoca Romania

**Keywords:** ammonia scavenging, AMPK signalling, hepatic fibrosis, L‐ornithine L‐aspartate, metabolic dysfunction‐associated steatotic liver disease, metabolic‐dysfunction associated steatohepatitis

## Abstract

**Introduction:**

While hyperammonemia is traditionally associated with decompensated cirrhosis, emerging evidence suggests that disturbances in nitrogen homeostasis contribute to disease progression in earlier stages of steatohepatitis and fibrosis. L‐ornithine L‐aspartate (LOLA), an established ammonia scavenger, targets key pathophysiological mechanisms shared by metabolic dysfunction‐associated steatotic liver disease (MASLD), including oxidative stress, mitochondrial dysfunction and hepatic stellate cell activation. This systematic review synthesizes current experimental and clinical research to evaluate the potential therapeutic role of LOLA in MASLD.

**Methods:**

A systematic search of PubMed, Embase and SCOPUS was conducted up to December 1, 2025, following PRISMA 2020 guidelines. Eligible studies included experimental (in vivo/in vitro) and clinical trials evaluating the effects of LOLA or L‐aspartate on hepatic steatosis, inflammation, or fibrosis in the context of MASLD. Data extraction and quality assessment were performed independently by two reviewers using appropriate tools for animal and human studies.

**Results:**

Nineteen studies were included, comprising 10 experimental pre‐clinical models (9 in vivo animal studies and 1 in vitro study) and 9 clinical studies involving approximately 1671 participants. Experimental studies consistently demonstrated that LOLA intervention ameliorates hepatic steatosis, inflammation and collagen deposition. Identified molecular mechanisms included the activation of the LKB1‐AMPK axis, restoration of mitochondrial bioenergetics and modulation of the gut‐liver‐muscle axis. In clinical studies, results from three randomized controlled trials (RCTs) indicated significant improvements in liver enzymes (ALT, AST) and lipid profiles, with reductions in hepatic steatosis. Evidence from six observational and open‐label studies corroborated these biochemical improvements and further demonstrated significant reductions in blood ammonia levels, improved intrahepatic microcirculation and reduced liver stiffness and patient‐reported fatigue. However, clinical evidence remains limited by study heterogeneity and a lack of large‐scale randomized trials using specific MASLD criteria.

**Conclusions:**

Preclinical evidence suggests that LOLA exerts pleiotropic hepatoprotective effects in MASLD by targeting hyperammonemia‐induced fibrosis and metabolic dysregulation. While growing clinical data indicate benefits in biochemical normalization, structural improvement and symptom relief, further robust clinical research is required to validate these findings and establish LOLA as a standard therapeutic option for MASLD patients.

## Graphical abstract

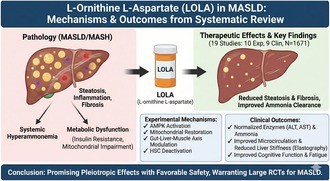

## 1 INTRODUCTION

Metabolic‐dysfunction associated steatotic liver disease (MASLD), formerly known as non‐alcoholic fatty liver disease (NAFLD), has emerged as a global epidemic, paralleling the rising prevalence of obesity and type 2 diabetes mellitus.^1^ Currently affecting approximately 30% of the global adult population, MASLD represents a spectrum of liver injury ranging from simple steatosis to metabolic‐dysfunction associated steatohepatitis (MASH), which can progress to fibrosis, cirrhosis and hepatocellular carcinoma.^2^ Recently, the nomenclature was updated from NAFLD/MAFLD to MASLD to more accurately reflect the underlying pathogenesis driven by metabolic dysregulation and to remove the stigmatization associated with earlier terminology.^3^, ^4^ Despite this shift in definition, the clinical imperative remains urgent: identifying therapeutic agents that can halt the progression from inflammatory steatohepatitis to irreversible fibrosis is a critical unmet need in modern hepatology.

The pathophysiology of MASLD is multifactorial, governed by a ‘multiple‐hit’ hypothesis involving insulin resistance, lipotoxicity, oxidative stress and mitochondrial dysfunction.^5^ However, recent experimental evidence suggests that disturbances in nitrogen homeostasis and the urea cycle play a pivotal, underappreciated role in disease progression.^6^ Even in non‐cirrhotic stages of MASLD, functional impairments in the urea cycle can lead to systemic hyperammonemia. This excess ammonia acts as a potent toxin, not only to the brain but also to the liver and skeletal muscle.^7^ Hyperammonemia activates hepatic stellate cells (HSCs), thereby driving fibrogenesis and inducing myostatin expression in skeletal muscle, contributing to sarcopenia.^8^ This ‘liver‐muscle axis’ deterioration creates a vicious cycle where sarcopenia further reduces the body's capacity to detoxify ammonia, exacerbating metabolic injury.^9^

Current pharmacological management of MASLD is limited, with lifestyle modification and weight loss remaining the cornerstone of therapy.^10^, ^11^ While agents such as resmetirom and semaglutide are currently recommended in advanced MASH fibrosis,^12^, ^13^ there is no universally adopted pharmacotherapy that targets the pleiotropic metabolic defects of the disease, particularly the ammonia‐related toxicity. L‐ornithine L‐aspartate (LOLA), a stable salt of two endogenous amino acids, is currently indicated primarily for the treatment of hepatic encephalopathy (HE) in cirrhosis.^14^ Its established mechanism involves the stimulation of the urea cycle in periportal hepatocytes via carbamoyl phosphate synthetase 1 (CPS1) and the induction of glutamine synthesis in perivenous hepatocytes and skeletal muscle, thereby effectively scavenging systemic ammonia.^15^

Given its unique mechanism of action, we hypothesize that LOLA may offer therapeutic benefits in MASLD beyond simple ammonia detoxification. By lowering ammonia levels, LOLA may deactivate HSCs and improve hepatic microcirculation, thereby attenuating fibrosis.^16^ Furthermore, the metabolism of LOLA generates glutamate, a precursor for glutathione (GSH), potentially restoring the oxidant/antioxidant balance and reducing lipid peroxidation in the steatotic liver.^17^ Additionally, by breaking the cycle of hyperammonemia‐induced muscle depletion, LOLA may address the sarcopenic obesity often observed in MASH patients.^18^ The repurposing of this established agent could represent a novel strategy to target the metabolic and fibrotic drivers of MASLD simultaneously.

Therefore, the aim of this study is to conduct a systematic review of existing experimental and clinical literature to evaluate the efficacy and multimodal mechanisms of LOLA in MASLD. Specifically, we aim to assess the impact of LOLA on key histological outcomes (steatosis and fibrosis), biochemical markers of liver injury (ALT, AST) and emerging metabolic targets, including mitochondrial bioenergetics, the LKB1‐AMPK axis and the gut‐liver‐muscle axis. By synthesizing data from animal models and human studies, this review seeks to determine whether LOLA intervention can ameliorate liver injury and halt fibrosis progression via both ammonia‐dependent and independent pathways, thereby providing an evidence base for its potential expanded use in the management of MASLD patients.

## 2 METHODS

This systematic review was conducted and reported in accordance with the guidelines set forth by the Preferred Reporting Items for Systematic Reviews and Meta‐Analyses (PRISMA) 2020 statement.^19^

### 2.1 Data sources and search strategy

To comprehensively identify relevant studies evaluating the efficacy and mechanisms of LOLA in MASLD, we conducted a systematic search of electronic databases including PubMed, Embase and SCOPUS from their inception through December 1, 2025. The search strategy employed a combination of controlled vocabulary and free‐text keywords to address the evolving nomenclature of the disease. Specifically, the intervention terms ‘ornithylaspartate’ or ‘LOLA’ were combined using Boolean operators with disease terms including ‘Non‐alcoholic Fatty Liver Disease,’ ‘MAFLD’ and ‘MASLD,’ alongside their variations such as ‘Metabolic dysfunction associated fatty liver disease’ and ‘Metabolic dysfunction associated steatotic liver disease.’ To ensure the inclusion of all pertinent literature and minimize selection bias, we also manually screened the reference lists of included articles and relevant reviews for additional eligible studies that may not have been captured by the electronic search.

### 2.2 Study selection and eligibility criteria

All experimental and clinical studies evaluating the role of LOLA or L‐aspartate in the context of fatty liver disease were considered for inclusion. Eligibility was determined based on strict inclusion criteria, selecting randomized controlled trials, observational clinical studies and experimental research using animal or cell culture models. We specifically included studies where hepatic steatosis or fibrosis was associated with metabolic dysfunction or toxic liver injury mimicking these pathologies, and where the intervention involved the administration of LOLA or L‐aspartate. Furthermore, studies were required to assess relevant outcomes such as histological changes, biochemical markers including liver enzymes and ammonia, or molecular mechanisms.

Conversely, we excluded studies that did not utilize LOLA or L‐aspartate, those focusing solely on hepatic encephalopathy without evaluating liver tissue or metabolic parameters related to steatosis or fibrosis, as well as duplicate data and non‐original research such as editorials, letters, reviews and conference abstracts. Screening of titles and abstracts was performed by two independent investigators to exclude irrelevant records (V.C. and A.I.), followed by a full‐text review of potentially eligible articles. Any discrepancies regarding study eligibility were resolved through consensus or consultation with a third investigator (D.L.D.).

### 2.3 Data extraction

Data from the included studies were extracted into a standardized electronic form by one investigator (V.C.) and subsequently verified for accuracy by a second reviewer (A.I.). The extracted variables included bibliometric details such as the first author, publication year and country of origin, alongside specific characteristics of the experimental model, including animal strains, diets used to induce MASLD, cell lines or human patient demographics. We detailed the intervention specifics, noting the specific agent used, dosage, route of administration and duration of treatment. Key outcomes were retrieved, encompassing histological improvements in steatosis grade and fibrosis stage, biochemical changes in ALT, AST, ammonia and lipid profiles, as well as molecular findings related to oxidative stress markers and gene expression. Additionally, proposed mechanisms of action, such as AMPK activation or urea cycle stimulation, and the primary conclusions of each study were synthesized.

### 2.4 Quality assessment

The methodological quality and potential risk of bias of the included studies were evaluated using tools appropriate for the specific study designs. Animal intervention studies were assessed using the SYRCLE's risk of bias tool to evaluate domains such as selection bias, performance bias and detection bias.^20^ For clinical research, quality was appraised using established scales, specifically the Newcastle‐Ottawa Scale for observational cohorts^21^ and the Cochrane Risk of Bias tool for randomized trials.^22^ This assessment focused on critical factors including the randomization process, comparability of groups, blinding of outcome assessment and the completeness of outcome data. Two reviewers independently conducted the quality assessment (V.C. and A.I.), with any disagreements resolved through discussion to achieve consensus.

## 3 RESULTS

### 3.1 Literature search

**FIGURE 1:**
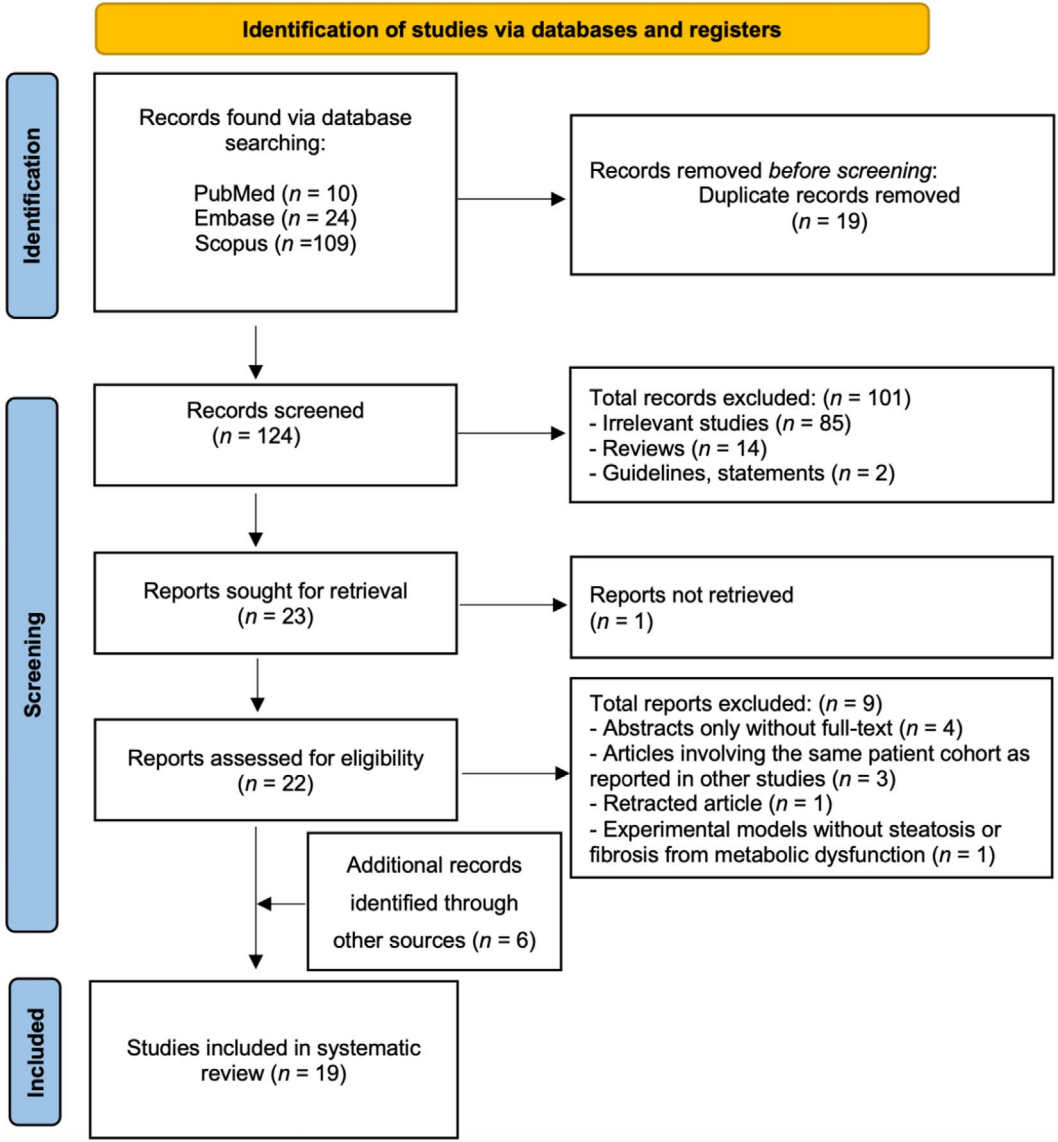
PRISMA flow diagram outlining the identification, screening and inclusion phases.

The systematic literature search identified a total of 143 records from the electronic databases, comprising 10 articles from PubMed, 24 from Embase and 109 from SCOPUS. Following the removal of 19 duplicates, the remaining 124 records were carefully reviewed through an assessment of titles and abstracts. A total of 101 records were excluded at this stage due to the following reasons: (1) irrelevant studies (*n* = 85); (2) review articles (*n* = 14); and (3) guidelines or consensus statements (*n* = 2). Of the 23 reports sought for retrieval, one could not be retrieved. The eligibility of the remaining 22 full‐text articles was further evaluated. During this phase, 9 records were excluded due to: (1) abstracts only without full text (*n* = 4)^23^, ^24^, ^25^, ^26^; (2) articles involving the same patient cohort as reported in other included studies (*n* = 3); (3) retracted article (*n* = 1)^27^; or (4) experimental models lacking steatosis or fibrosis associated with metabolic dysfunction (*n* = 1).^28^ Additionally, manual screening of reference lists and specialized sources identified 6 further eligible studies. Consequently, a total of 19 studies, comprising 10 experimental animal or in vitro models^28^, ^29^, ^30^, ^31^, ^32^, ^33^, ^34^, ^35^, ^36^ and 9 clinical studies,^37^, ^38^, ^39^, ^40^, ^41^, ^42^, ^43^, ^44^, ^45^ fulfilled our criteria and were included in this systematic review, as described in Figure 1.

### 3.2 Study characteristics

The main characteristics of the included studies are summarized in Tables 1 and 2. A total of 19 studies were included in this systematic review, comprising both experimental models and clinical investigations. Ten studies employed experimental designs, utilizing animal models of liver injury, specifically C57BL/6 mice^28^, ^29^, ^30^, ^31^, ^32^ and Wistar or Sprague Dawley rats,^33^, ^34^, ^35^, ^36^ or in vitro human hepatocyte cultures.^46^ Nine studies were conducted in a clinical setting, consisting of one large‐scale observational study^37^ and eight prospective clinical trials.^38^, ^39^, ^40^, ^41^, ^42^, ^43^, ^44^, ^45^

**TABLE 1:** Experimental studies evaluating the role of LOLA in fatty liver disease.

First author (Year)/Country	Experimental model	Intervention details (Agent/Dose/Duration)	Key histological & biochemical outcomes	Proposed mechanism/pathway	Key conclusion
Prikhodko et al. (2020)^30^/Russia	Mice (C57BL/6) NAFLD model induced by high‐caloric ‘Western’ diet + CCl_4_ injections for 6 months	LOLA (Hepa‐Merz) 1.5 g/kg/day orally (intragastric)	Physical Performance: Significantly increased forced swim time (+106% vs. control) and exhaustive swim endurance. Recovery: Improved post‐exercise recovery rates.	Ammonia Detoxification & Bioenergetics: Reduction of hyperammonemia‐induced physical fatigue (asthenic syndrome) and improvement of muscle bioenergetics via the urea cycle and glutamine synthesis.	LOLA significantly improves physical work capacity and accelerates post‐exercise recovery in animals with experimental NAFLD/steatohepatitis.
Rao et al. (2021)^31^/China	Mice (C57BL/6) HFC diet‐induced obese MAFLD	L‐aspartate 200 mg/kg/day orally	Decreased hepatic TG and DAG. Reduced ALT, AST, ALP. Ameliorated steatosis and inflammation.	LKB1‐AMPK Axis: L‐aspartate activates the LKB1‐AMPK axis, stimulating mitochondrial lipid oxidation and improving gut‐liver bile acid metabolism.	L‐aspartate mimics the beneficial metabolic effects of *A. muciniphila*, reducing hepatic steatosis and injury by enhancing lipid oxidation.
Oleshchuk et al. (2021)^36^/Ukraine	Rats (Wistar) Toxic hepatitis (CCl_4_ induced)	LOLA 200 mg/kg i.p. for 7 days	Restored liver structure. Reduced ALT, AST, ALP, GGT. Increased total protein and urea. Decreased TBARS; increased Catalase/SOD/GSH.	Antioxidant & Urea Cycle: Preserves oxidant/antioxidant balance (GSH production) and prevents inhibition of urea synthesis.	LOLA prevents cytolysis, cholestasis and oxidative damage in toxic liver injury, improving metabolic function.
Pichon et al. (2022)^29^/Belgium	Mice (*foz/foz* Alms1 mutant) HFD‐induced NASH	LOLA 2 g/kg/day in drinking water for 8 weeks	Prevented myosteatosis (muscle fat). No significant impact on liver histology (steatosis/fibrosis/inflammation) or plasma ammonia in this specific model.	Muscle Quality Preservation: Direct beneficial impact on skeletal muscle preventing fat infiltration but failed to restore hepatic ammonia detoxification efficiency in this genetic model.	LOLA prevented myosteatosis and maintained muscle quality during NASH development but did not improve liver disease or hyperammonemia in *foz/foz* mice.
de Freitas et al. (2022)^33^/Brazil	Rats (Sprague Dawley) HFCD diet (NAFLD model)	LOLA 200 mg/kg/day gavage (alone or with Vit E)	LOLA alone decreased miR‐122, miR‐33a, miR‐186. LOLA + VitE reduced atherogenic ratios and prevented collagen deposition.	Paracrine Signalling (miRNA): Modulation of microRNAs related to lipid metabolism, inflammation and endothelial dysfunction.	LOLA (especially combined with Vit E) improves atherogenic dyslipidemia, fibrosis and paracrine signalling in NAFLD.
Canbay et al. (2024)^46^/Germany	In Vitro/Ex Vivo Human Hepatocytes & HepG2 cells (Steatosis/Insulin Resistance models)	LOLA Concentration‐dependent (up to 40 mM)	Reduced cellular NH3 release. Downregulated fatty acid transport (*CD36*) and synthesis genes (*FASN*, *SREBF1*). Reconstituted mitochondrial membrane potential.	Energy Metabolism: Reduces cellular ATP and acetyl‐CoA; activates AMPK‐α; modulates lipid transport genes; prevents mitochondrial depolarization.	LOLA normalizes metabolic parameters, restores mitochondrial integrity and acts on fatty acid transport/synthesis targets, suggesting potential for NAFLD treatment.
Guo et al. (2024)^28^/China	Mice (C57BL/6) HFC diet‐induced obesity	L‐aspartate 200 mg/kg/day orally	Reduced body weight (8.5%–14.5% reduction) and adipose tissue weight. Ameliorated hepatocyte ballooning and lipid accumulation.	Energy Expenditure (Adipocytes): Increases whole‐body energy expenditure; suppresses adipogenesis/lipogenesis via activation of the AMPK signalling pathway.	L‐aspartate is an endogenous inducer of energy expenditure that ameliorates diet‐induced obesity and metabolic syndrome.
Lange et al. (2024)^34^/Brazil	Rats (Sprague Dawley) HFCD diet (MASLD model)	LOLA 200 mg/kg/day gavage	Altered specific gut microbes (*Helicobacter rodentium*, *Parabacteroides*). No change in alpha/beta diversity.	Microbiota‐Metabolism Axis: Modulates metabolic pathways related to L‐aspartate (TCA cycle, nucleotide biosynthesis) rather than L‐ornithine pathways.	LOLA influences specific probiotic gut microbes and metabolic profiles linked to L‐aspartate, despite not changing overall diversity.
Longo et al. (2024)^35^/Brazil	Rats (Sprague Dawley) HFCD diet (MASLD model)	LOLA 200 mg/kg/day gavage	Diminished collagen deposition. Reduced oxidative stress markers (carbonyl, TBARS). Normalized Type 3 Deiodinase (D3) levels.	Thyroid Hormone & ER Stress: Regulates Type 3 deiodinase (D3), improves Krebs cycle enzyme activity (SDH, GDH) and reduces endoplasmic reticulum stress (GRP78).	LOLA improves energy production, thyroid hormone metabolism and oxidative stress, likely via GSH replenishment from the glutamate portion.
Su et al. (2024)^32^/Brazil	Mice (C57BL/6) CCl_4_‐induced fibrosis	L‐aspartate 100 mg/kg orally every other day	Alleviated liver injury (AST, ALT) and fibrosis (Collagen I, alpha‐SMA). Reduced hepatic corticosterone (CORT) levels.	GR beta Signalling: Reverses CORT‐mediated Glucocorticoid Receptor beta (GR beta) mitochondrial malfunction; rebalances cholesterol‐steroid metabolism.	L‐aspartate ameliorates fibrosis by suppressing GR beta signalling and rebalancing hepatic cholesterol‐steroid metabolism.

Abbreviations: AMPK, AMP‐activated protein kinase; D3: Type 3 Deiodinase; GR beta: Glucocorticoid Receptor Beta; GSH: Glutathione; HFC/HFCD: High‐Fat High‐Cholesterol/High‐Fat Choline‐Deficient; TBARS: Thiobarbituric acid reactive substances; TG/DAG: Triglycerides/Diacylglycerol.

**TABLE 2:** Original Clinical Studies Evaluating the Role of LOLA in Fatty Liver Disease.

First Author (Year) [Country]	Experimental model	Intervention details (Agent/Dose/Duration)	Key histological & biochemical outcomes	Proposed Mechanism/Pathway	Key conclusion
Grüngreiff et al. (2001)^37^/Germany	Humans (Observational) 1167 patients with chronic liver disease (648 fatty liver, 378 cirrhosis, 253 chronic hepatitis)	LOLA (Granules) Oral administration Variable doses (typically 6–9 g/day) Duration: 30–90 days	Biochemical: Significant dose‐dependent reduction in liver enzymes (AST, ALT, GGT) by ~40%–50%. Clinical: Improvement in fatigue, sleep disorders and concentration.	Ammonia Detoxification: Stimulates urea and glutamine synthesis; promotes antioxidant glutathione production.	LOLA is effective and well‐tolerated for symptomatic treatment and enzyme reduction in chronic liver disease, particularly fatty liver.
Tian et al. (2013)^43^/China	Humans (RCT) 72 patients with NASH	LOLA (Granules) Oral administration Group A: 6 g/day Group B: 3 g/day Duration: 12 weeks	Biochemical: Significant reduction in ALT and Triglycerides (TG) in the high‐dose group. Imaging: Significant improvement in liver/spleen CT ratio in the high‐dose group.	Lipid Metabolism & Hepatoprotection: Reduction of liver enzymes and triglycerides suggests improved hepatic lipid metabolism and reduced inflammation.	High‐dose (6 g/day) LOLA significantly improves liver enzymes and reduces hepatic fat accumulation in NASH patients.
Zhuravleva et al. (2015)^45^/Ukraine	Humans (Clinical) 52 obese patients with NAFLD and Metabolic Syndrome (Group A: Diet only; Group B: Diet + LOLA)	LOLA (Larnamin) Oral administration 9 g/day Duration: 6 months	Biochemical: Normalization of ALT, GGT, CRP and lipid profile (TG, LDL); improved insulin sensitivity (HOMA‐IR). Imaging: Significant reduction in liver size and echogenicity (steatosis). Clinical: Improved weight loss and fat distribution compared to diet alone.	Metabolic Regulation: LOLA enhances the effects of hypocaloric diet by correcting insulin resistance, lipid metabolism and systemic inflammation (CRP).	Long‐term LOLA therapy combined with diet significantly reduces steatosis, inflammation and metabolic syndrome components in obese NAFLD patients.
Ilchenko et al. (2016)^38^/Russia	Humans (RCT) 30 patients with NAFLD or Alcoholic Fatty Liver Disease (Steatosis/Steatohepatitis)	LOLA (Hepa‐Merz) Oral administration 9 g/day Duration: 4 weeks	Biochemical: Reduction in ALT, AST, GGT and bilirubin. Clinical: Improvement in cognitive function (NCT) and quality of life (CLDQ); reduction in asthenia and dyspepsia.	Ammonia Detoxification & Gut‐Liver Axis: Reduction of ammonia improves cognitive function (minimal HE) and liver function; combination with probiotics showed additive benefits.	LOLA is effective as monotherapy or in combination with probiotics for improving liver function, cognitive status and quality of life in fatty liver disease.
Ageeva et al. (2017)^39^/Russia	Humans (Clinical) 37 patients with chronic liver disease (21 Fatty Liver, 16 HCV) at pre‐cirrhotic stage with hyperammonemia	LOLA (Oral) 9 g/day Duration: 4 weeks (Course 1); then 10 days/month for 3 months (Course 2)	Biochemical: Significant reduction in blood ammonia levels (from 56.1 to 34.7 μmol/L). Long‐term: 67.6% maintained normal ammonia at 6 months; recurrent hyperammonemia in 32.4% was effectively treated with intermittent courses.	Ammonia Scavenging: Correction of hyperammonemia at the pre‐cirrhotic stage to prevent progression of portal hypertension and fibrosis.	Oral LOLA effectively reduces hyperammonemia in pre‐cirrhotic stages of liver disease, including fatty liver, preventing potential fibrotic progression.
Sas (2018)^44^/Russia	Humans (Clinical) 96 patients with NASH	LOLA (Hepa‐Merz) Oral administration 3 g/day Duration: 2 months	Biochemical: Significant reduction in ALT, AST and improvement in lipid profile (Total Cholesterol, LDL, TG). Hormonal: Significant increase in Somatotropic Hormone (STH) levels.	Hormonal & Anabolic Effect: LOLA stimulates STH secretion, promoting protein synthesis and lipolysis, counteracting metabolic syndrome features.	LOLA improves liver enzymes and atherogenic dyslipidemia in NASH, potentially mediated by increased growth hormone levels and anabolic effects.
Kizova et al. (2019)^42^/Russia	Humans (Clinical) 103 patients with NAFLD (pre‐cirrhotic)	LOLA (Hepa‐Merz) Oral administration 9 g/day Duration: 4 weeks	Biochemical: Significant reduction in blood ammonia levels. Clinical: Significant improvement in Number Connection Test (NCT) time (cognitive function).	Ammonia Detoxification: Reduction of subclinical hyperammonemia improves cognitive performance and may prevent neurotoxic and hepatotoxic effects.	LOLA effectively reduces ammonia and improves cognitive function in pre‐cirrhotic NAFLD patients, highlighting the prevalence of hyperammonemia in this population.
Ermolova et al. (2020)^23^, ^40^/Russia	Humans (Clinical) 69 patients (43 NASH, 26 HCV) with early fibrosis (F0–F2)	LOLA (Ornithine) Oral administration 9 g/day Duration: 4 weeks	Biochemical: Significant reduction in blood ammonia. Hemodynamics: Improved intrahepatic microcirculation. Histology: Baseline biopsies showed HSC activation (alpha‐SMA).	HSC Deactivation & Microcirculation: LOLA lowers ammonia, thereby deactivating Hepatic Stellate Cells (HSCs) and restoring intrahepatic blood flow.	LOLA reduces hyperammonemia and improves intrahepatic hemodynamics in early‐stage NASH, potentially slowing fibrogenesis.
Garanina (2021)^41^/Russia	Humans (Clinical) 45 patients with MAFLD (Steatosis S2–S3, Fibrosis F1) and hyperammonemia	LOLA Oral administration 9 g/day Duration: 8 weeks	Biochemical: Normalized ammonia; reduction in ALT, AST, GGT, Ferritin, CRP. Fibrosis/Steatosis: Reduced liver stiffness (FibroScan) in 85% and steatosis grade (CAP) in 69% of patients.	Ammonia Scavenging & Anti‐inflammatory: Targeting hyperammonemia reduces ‘second hit’ hepatotoxicity and systemic inflammation, dampening fibrosis.	LOLA normalizes ammonia and significantly reduces inflammation, steatosis and fibrosis stiffness in MAFLD patients.

Abbreviations: alpha‐SMA, Alpha‐Smooth Muscle Actin; ALT, Alanine Aminotransferase; AST, Aspartate Aminotransferase; CAP, Controlled Attenuation Parameter; CLDQ, Chronic Liver Disease Questionnaire; CRP, C‐Reactive Protein; CT, Computed Tomography; GGT, Gamma‐glutamyl Transferase; HCV, Hepatitis C Virus; HE, Hepatic Encephalopathy; HOMA‐IR, Homeostatic Model Assessment for Insulin Resistance; HSC, Hepatic Stellate Cell; LDL, Low‐Density Lipoprotein; LOLA, L‐Ornithine L‐Aspartate; MAFLD, Metabolic‐Associated Fatty Liver Disease; NAFLD, Non‐Alcoholic Fatty Liver Disease; NASH, Non‐Alcoholic Steatohepatitis; NCT, Number Connection Test; RCT, Randomized Controlled Trial; STH, Somatotropic Hormone; TG, Triglycerides.

The clinical studies involved a total of approximately 1671 human subjects. The specific patient populations varied, ranging from 30 patients with steatosis or^38^ to a large cohort of 1167 patients with various chronic liver diseases, including fatty liver and hepatitis.^37^ Regarding geographical distribution, the studies were conducted across three continents: four studies in Asia (China),^28^, ^31^, ^32^, ^43^ twelve in Europe,^30^ Russia,^38^, ^39^, ^40^, ^41^, ^42^, ^44^ Germany,^37^, ^46^ Ukraine^36^, ^45^ and Belgium^29^ and three in South America (Brazil).^33^, ^34^, ^35^

### 3.3 Definition and induction of hepatic steatosis and fibrosis

In the experimental studies included, hepatic steatosis and fibrosis were induced using established dietary or chemical models to mimic the pathophysiology of MASLD. The majority of animal studies (*n* = 6) utilized dietary interventions, specifically High‐Fat Diets (HFD), High‐Fat High‐Cholesterol (HFC) diets or High‐Fat Choline‐Deficient (HFCD) diets to generate phenotypes of steatosis and non‐alcoholic steatohepatitis (NASH).^28^, ^29^, ^31^, ^33^, ^34^, ^35^ Two studies employed chemical induction via carbon tetrachloride (CCl_4_) to establish models of toxic hepatitis and liver fibrosis,^32^, ^36^ while one study utilized a combination of a high‐caloric ‘Western’ diet and CCl_4_ injections.^30^ Additionally, one in vitro study modelled steatosis by exposing human hepatocytes to free fatty acids (oleate: palmitate).^46^

In the clinical studies, the definition and evaluation of liver disease severity varied by methodology. One study utilized transient elastography (FibroScan®) with Controlled Attenuation Parameter (CAP) measurement to non‐invasively quantify liver stiffness (fibrosis) and steatosis grades.^41^ Another study relied on liver biopsy and histological assessment to determine fibrosis stages according to the METAVIR scoring system.^40^ Several prospective trials evaluated steatosis and fibrosis using non‐invasive methods such as ultrasound, computed tomography and biochemical markers.^38^, ^39^, ^42^, ^43^, ^44^, ^45^ The observational cohort study defined fatty liver and chronic hepatitis based on established clinical diagnoses and enzyme elevations.^37^

### 3.4 Experimental studies: Mechanisms of hepatoprotection and metabolic regulation

In diverse pre‐clinical models of liver injury, the administration of LOLA or L‐aspartate consistently ameliorated hepatic steatosis and inflammation, evidenced by significant reductions in hepatic triglycerides and serum transaminases.^28^, ^31^, ^36^ Specifically, in a rat model of toxic liver injury, LOLA treatment reduced serum ALT and AST levels by approximately 35% and 40%, respectively, while significantly normalizing serum albumin and bilirubin levels compared to untreated controls.^36^ Several studies highlighted the restoration of mitochondrial function and activation of the AMPK signalling pathway as central mechanisms driving lipid oxidation and energy expenditure.^32^, ^35^, ^46^ For instance, L‐aspartate administration was shown to upregulate the phosphorylation of AMPK and Acetyl‐CoA Carboxylase (ACC), resulting in a marked decrease in lipid droplet accumulation in hepatocytes and a restoration of mitochondrial membrane potential.^31^, ^46^ Furthermore, interventions targeting the gut‐liver axis demonstrated that LOLA modulates the microbiome and bile acid metabolism,^31^, ^34^ while others established a critical link between ammonia scavenging and the preservation of skeletal muscle mass and function in sarcopenic obesity.^29^ Notably, LOLA supplementation enriched the abundance of beneficial *Parabacteroides* species and modulated nucleotide biosynthesis pathways, which correlated with improved hepatic lipid profiles.^34^ In the context of physical performance, LOLA was also shown to improve muscle bioenergetics and recovery, countering the asthenic syndrome associated with experimental steatohepatitis.^30^ Experimental data indicated that LOLA increased the duration of forced swimming by 106% and accelerated the restoration of physical capacity during the post‐exercise recovery period.^30^ Finally, distinct anti‐fibrotic effects were observed through the suppression of hepatic stellate cell activation and modulation of glucocorticoid receptor signalling.^32^, ^33^ This was quantitatively demonstrated by a significant downregulation of *α*‐smooth muscle actin (*α*‐SMA) expression and a reduction in collagen deposition in liver tissue samples, alongside the reversal of glucocorticoid receptor *β*‐mediated mitochondrial dysfunction.^32^, ^33^

### 3.5 Original clinical studies: Biochemical normalization and structural improvement

In clinical settings, oral supplementation with LOLA demonstrated significant efficacy in normalizing biochemical profiles, specifically reducing blood ammonia and liver enzymes in patients with chronic liver diseases and metabolic dysfunction‐associated fatty liver disease.^37^, ^38^, ^39^, ^40^, ^41^, ^42^, ^43^, ^44^, ^45^ Specifically, clinical trials reported a significant reduction in serum ALT levels by approximately 40%–50% from baseline, with one study showing a decrease in blood ammonia from 56.1 μmol/L to 34.7 μmol/L after 4 weeks of treatment.^37^, ^38^ Beyond biochemical improvements, LOLA treatment was associated with structural and hemodynamic benefits, including significant reductions in liver stiffness and steatosis grades measured by transient elastography^41^ and the restoration of intrahepatic microcirculation via the deactivation of hepatic stellate cells.^40^ In a cohort of 45 MAFLD patients, liver stiffness decreased significantly in 85% of subjects, while Controlled Attenuation Parameter (CAP) scores indicative of steatosis grade improved in 69% of patients following an 8‐week course of LOLA.^41^ Large‐scale observational data further supported these findings, reporting dose‐dependent improvements in clinical symptoms such as fatigue and cognitive deficits alongside enzyme reduction.^37^, ^38^, ^42^ In a large observational study of 1167 patients, efficacy was rated as ‘very good’ or ‘good’ by 90% of physicians, correlating with a notable improvement in fatigue scores and concentration.^37^

### 3.6 Impact on extra‐hepatic manifestations: Sarcopenia and the gut microbiome

Several studies extended their investigation beyond the liver to assess the systemic effects of LOLA, particularly on the gut‐liver‐muscle axis. In a genetic model of NASH (*foz*/*foz* mice), preventative LOLA treatment successfully inhibited the development of myosteatosis (muscle fat infiltration) and preserved muscle quality, although it did not reverse established sarcopenia in the therapeutic setting.^29^ Preventative treatment significantly reduced intramuscular lipid droplet size and maintained muscle mass at levels comparable to healthy controls, whereas therapeutic intervention only marginally affected myofiber diameter.^29^ Functional improvements were also observed in experimental models, where LOLA significantly increased physical work capacity and accelerated post‐exercise recovery.^30^ Specifically, exhaustive swim endurance in LOLA‐treated animals increased by over 100% compared to the placebo group, with a concomitant reduction in post‐exercise lactate accumulation.^30^ These findings were mirrored in clinical settings, where long‐term LOLA administration combined with a hypocaloric diet resulted in a significant increase in relative muscle mass compared to diet alone.^45^ Over a 6‐month period, patients receiving LOLA exhibited a greater improvement in anthropometric muscle indices and a more favourable fat‐to‐muscle mass ratio than those on dietary restriction alone.^45^

Regarding the gut microbiome, L‐aspartate was shown to mimic the beneficial metabolic effects of *Akkermansia muciniphila* by facilitating the transport of L‐aspartate from the gut to the liver, thereby improving bile acid metabolism.^31^ Mechanistically, L‐aspartate supplementation upregulated the expression of hepatic bile acid synthesis enzymes (CYP7A1) and promoted the faecal excretion of secondary bile acids.^31^ Furthermore, LOLA treatment in rats induced specific compositional changes in the intestinal microbiota, altering the abundance of probiotic species such as *Parabacteroides* and modulating metabolic pathways related to nucleotide biosynthesis.^34^ Quantitative 16S rRNA analysis revealed a significant enrichment in Bacteroidetes and a reduction in the Firmicutes/Bacteroidetes ratio, a marker often elevated in metabolic disorders.^34^ Clinical evidence further supports targeting this axis, with studies demonstrating that combining LOLA with probiotics can synergistically improve clinical symptoms and quality of life in patients with fatty liver disease.^38^ Patients treated with the combination therapy showed a significantly greater improvement in Chronic Liver Disease Questionnaire (CLDQ) scores, particularly in domains related to abdominal symptoms and fatigue, compared to monotherapy.^38^

### 3.7 Safety and tolerability

Data regarding the safety profile of LOLA were primarily derived from the large‐scale observational study included in this review. In a cohort of 1167 patients with chronic liver diseases, oral LOLA granules demonstrated a favourable safety profile, with adverse drug reactions reported in only 1.6% of the total population.^37^ The most common adverse events were mild gastrointestinal symptoms, such as nausea (<1%) and bloating, which resolved without discontinuation of therapy.^37^ The tolerability was rated as ‘very good’ or ‘good’ by the vast majority of participating physicians, supporting its viability for long‐term symptomatic treatment in patients with fatty liver and chronic hepatitis.^37^ Specifically, 97% of physicians rated global tolerability as ‘good’ or ‘very good’ after observing patients for an average duration of 3 months.^37^ Consistent with these findings, multiple prospective clinical trials reported no significant safety concerns or treatment‐related withdrawals.^38^, ^39^, ^40^, ^41^, ^42^, ^43^, ^45^ In a study involving 96 patients with NASH, no severe adverse events were recorded, although mild heartburn requiring symptom management was noted in a small minority of cases.^44^ Only 3 out of 96 patients (3.1%) reported transient heartburn, which was managed effectively with antacids and did not require cessation of LOLA treatment.^44^

### 3.8 Quality assessment

The summary of the conducted quality assessment results is outlined in Tables S1–S3.

#### 3.8.1 Experimental studies

The methodological quality of the 10 included experimental studies was assessed using the SYRCLE Risk of Bias tool. Overall, the studies demonstrated a low risk of bias in several key domains. All in vivo studies (10/10) reported adequate sequence generation, typically by randomizing animals into treatment groups,^28^, ^29^, ^30^, ^31^, ^32^, ^33^, ^34^, ^35^, ^36^ and ensured comparable baseline characteristics (e.g. weight, age) between groups at the start of the experiments.^28^, ^29^, ^30^, ^32^, ^33^, ^34^, ^35^, ^36^ Furthermore, most animal studies (9/10) explicitly mentioned maintaining controlled housing conditions to minimize environmental confounders.^28^, ^29^, ^30^, ^31^, ^32^, ^33^, ^34^, ^35^ The risk of incomplete outcome data and selective reporting was consistently low across all studies, as protocols and outcomes were clearly defined and fully reported.

However, significant gaps in reporting were identified in domains related to blinding and allocation concealment. The majority of studies were rated as having an ‘unclear’ risk of bias for allocation concealment, as specific methods used to conceal the allocation sequence were rarely described. Similarly, blinding of caregivers/investigators during the intervention phase was frequently unreported (unclear risk). While several studies explicitly mentioned blinding during outcome assessment (e.g. histological analysis performed by a blinded pathologist or automated quantification),^29^, ^31^, ^33^, ^35^ others did not provide sufficient detail. The in vitro study by Canbay et al. was assessed only on applicable domains, showing low risk for selective reporting and incomplete data, but unclear risk for blinding.^46^ These findings suggest that while the experimental design and reporting of results are generally robust, future preclinical research should adhere more strictly to reporting guidelines (such as ARRIVE) regarding blinding and allocation concealment to further reduce potential bias.

#### 3.8.2 Clinical studies

The methodological quality of the nine included clinical studies was evaluated using tools appropriate for their respective designs. The three randomized controlled trials (RCTs) generally demonstrated a low risk of bias regarding random sequence generation, as all utilized randomization procedures to assign treatment arms.^38^, ^43^, ^45^ However, allocation concealment was frequently rated as ‘unclear’ due to a lack of specific details on how the randomization sequence was protected.^38^, ^45^ Regarding blinding, while Tian et al. utilized a controlled design with potential blinding of assessors,^43^ the other trials were open‐label, introducing a high risk of performance bias, although the objective nature of biochemical outcomes (e.g. liver enzymes, ammonia) likely mitigates detection bias.^38^, ^45^

For the six observational and non‐randomized studies, the Newcastle‐Ottawa Scale (NOS) revealed a generally ‘Good’ to ‘Fair’ quality.^37^, ^39^, ^40^, ^41^, ^42^, ^44^ Most studies scored well in the Selection domain, recruiting representative patient cohorts with clear diagnoses of liver disease.^37^, ^39^, ^40^, ^41^ However, the Comparability domain was a common source of weakness; while some studies included control groups or adjusted for baseline characteristics,^40^, ^41^ others lacked a non‐exposed control group, relying instead on before‐after comparisons, which limited their score.^42^, ^44^ Despite these limitations, the Outcome assessment was robust across all studies, utilizing standardized biochemical assays and validated diagnostic tools (e.g. FibroScan) with adequate follow‐up periods to detect treatment effects.^37^, ^41^

## 4 DISCUSSION

MASLD has emerged as a preeminent global health challenge, driving a significant burden of hepatic decompensation and extra‐hepatic morbidity.^47^ The pathophysiology of MASLD is intricate, involving a ‘multiple‐hit’ interplay of insulin resistance, lipotoxicity and oxidative stress.^5^ However, the contribution of nitrogen homeostasis dysregulation to disease progression remains underappreciated in mainstream hepatology.^48^ To our knowledge, this is the first systematic review to synthesize the full spectrum of translational evidence, spanning in vitro mechanistic data, animal models and clinical trials, evaluating LOLA in MASLD. By analysing 19 studies comprising 10 experimental models and 9 clinical trials with approximately 1671 participants, we provide robust evidence that LOLA exerts pleiotropic therapeutic effects. Our findings indicate that LOLA intervention not only normalizes biochemical markers of liver injury but also structurally ameliorates steatosis and fibrosis, challenging the traditional confinement of this agent to the treatment of hepatic encephalopathy.

Current literature increasingly identifies hyperammonemia not merely as a consequence of end‐stage cirrhosis, but as an active driver of fibrogenesis in earlier stages of liver disease.^8^ Recent transcriptomic analyses have shown that ammonia‐induced stress responses are upregulated in patients with steatohepatitis even in the absence of cirrhosis, correlating with disease severity.^49^ Our review substantiates this paradigm shift. Experimental data included in our analysis demonstrate that hyperammonemia directly activates HSCs, promoting their contraction and collagen production. Specifically, LOLA treatment was shown to reduce hepatic collagen area by approximately 50% in CCl4‐induced fibrosis models, a histological improvement directly linked to the downregulation of profibrotic markers such as TGF‐β 1 and TIMP‐1.^32^ By scavenging systemic ammonia, LOLA facilitates HSC deactivation, thereby improving intrahepatic microcirculation and halting fibrogenesis. This mechanism is clinically corroborated by significant reductions in liver stiffness measured by transient elastography in MAFLD patients. In a clinical cohort, 8 weeks of LOLA therapy resulted in a significant decrease in liver stiffness from 7.2 kPa to 5.6 kPa, suggesting a tangible regression of fibrotic burden or associated edema.^41^

Furthermore, our review uncovers novel metabolic targets of LOLA that extend beyond the urea cycle. We identified that L‐aspartate acts as an agonist for the LKB1‐AMPK axis, a master regulator of cellular energy homeostasis.^50^ Activation of this pathway stimulates mitochondrial fatty acid oxidation and suppresses de novo lipogenesis, effectively mimicking a ‘fasting state’ at the cellular level. This was evidenced by a significant reduction in hepatic triglyceride content and lipid droplet size in high‐fat diet‐fed mice treated with L‐aspartate.^31^ This is complemented by findings that LOLA restores mitochondrial membrane potential and reduces oxidative stress, addressing the mitochondrial dysfunction central to MASH pathogenesis.^51^ Mechanistically, LOLA has been shown to prevent the collapse of the mitochondrial membrane potential (ΔΨm) and reduce lipid peroxidation products like malondialdehyde in hepatocytes exposed to fatty acids.^14^, ^46^, ^52^ Additionally, recent studies have highlighted LOLA's ability to modulate the gut‐liver axis by altering the microbiome composition and to downregulate the glucocorticoid receptor beta signalling pathway, a novel fibrotic mediator.^53^

Our findings significantly expand upon the traditional understanding of LOLA, which has historically been confined to the management of hepatic encephalopathy (HE) in decompensated cirrhosis.^14^ While previous systematic reviews and clinical guidelines have firmly established LOLA's efficacy in lowering ammonia to ameliorate neurotoxicity,^54^ our review is among the first to systematically consolidate evidence supporting its direct hepatoprotective and anti‐steatotic effects in the pre‐cirrhotic stages of MASLD. Contrary to earlier paradigms that viewed ammonia primarily as a neurotoxin, the experimental data synthesized here align with emerging concepts of ammonia as a metabolic toxin that actively drives hepatic stellate cell activation and fibrosis.^55^ Furthermore, while existing literature focuses on the urea cycle, our review identifies the activation of the LKB1‐AMPK axis and the modulation of the gut‐liver axis as distinct,^56^ novel mechanisms by which LOLA combats lipotoxicity. This diverges from standard antioxidant therapies (e.g. Vitamin E), suggesting that LOLA offers a more comprehensive metabolic correction by simultaneously addressing nitrogen homeostasis, mitochondrial function and sarcopenic obesity.

A critical clinical implication of this review is the therapeutic relevance of LOLA in managing sarcopenic obesity, a phenotype associated with poor prognosis in MASLD.^57^ While emerging pharmacotherapies like GLP‐1 receptor agonists effectively reduce weight, they are often associated with the loss of lean muscle mass.^57^ In contrast, our analysis reveals that LOLA preserves skeletal muscle mass and function by providing substrates for glutamine synthesis and detoxification, thereby breaking the vicious cycle of hyperammonemia‐induced sarcopenia. In animal models of sarcopenic obesity, LOLA administration significantly increased myofiber cross‐sectional area and grip strength, directly countering the myostatin upregulation induced by hyperammonemia.^29^ Clinical data confirm that combining LOLA with hypocaloric diets improves body composition and relative muscle mass more effectively than diet alone. Patients receiving LOLA alongside lifestyle modification achieved a 4.2% increase in muscle mass percentage, compared to only 1.1% in the diet‐only group.^45^ This suggests a unique clinical positioning for LOLA, potentially as an adjunct therapy to preserve muscle health in patients undergoing aggressive weight loss regimens.

This review possesses several distinctive strengths that enhance the validity and clinical relevance of its findings. First, it represents the most comprehensive translational synthesis to date, effectively bridging the gap between intricate molecular mechanisms, such as microRNA modulation and AMPK activation, and tangible clinical outcomes, including quality of life and cognitive function. Second, the robustness of LOLA's therapeutic potential is underscored by the inclusion of diverse experimental models, ranging from high‐fat diet‐induced obesity to chemical fibrosis induction. This variety mimics the heterogeneous aetiology of human MASLD, suggesting that LOLA's efficacy is not limited to a single pathogenic pathway. Third, our analysis encompasses a substantial clinical cohort of approximately 1671 participants across multiple continents (Europe, Asia, South America), providing a degree of external validity often lacking in smaller, single‐center studies. Finally, by framing the results within the context of the updated MASLD nomenclature and the gut‐liver‐muscle axis, this review offers a modern, holistic perspective that supports the repurposing of an established, safe therapeutic agent for a growing global epidemic.

However, several limitations must be acknowledged. The clinical evidence base, while promising, is characterized by heterogeneity in study design. Due to the significant heterogeneity among the included studies, spanning diverse designs (from in vitro and animal models to observational cohorts and randomized trials), interventions and outcome measures, a quantitative meta‐analysis was not feasible. Therefore, a qualitative narrative synthesis of the evidence was performed. Many of the included clinical studies are open‐label or observational,^38^ which introduces a risk of selection and performance bias. Furthermore, most clinical trials utilized surrogate endpoints for fibrosis (e.g. elastography, biochemical scores) rather than the gold standard of paired liver biopsies. The duration of treatment in most studies was relatively short (4–12 weeks), leaving the long‐term impact of LOLA on hard endpoints, such as the prevention of hepatocellular carcinoma or decompensation, unknown. Finally, the variability in dosing regimens and the specific definitions of fatty liver disease (NAFLD vs. MAFLD vs. MASLD) across studies complicates the establishment of a standardized clinical protocol.

The evolution of nomenclature from NAFLD to MASLD emphasizes metabolic dysfunction, aligning perfectly with LOLA's mechanism of metabolic correction. Future research must prioritize large‐scale, double‐blind, randomized controlled trials utilizing the standardized MASLD criteria to rigorously evaluate efficacy. These trials should aim to include histological endpoints to definitively prove fibrosis regression. Additionally, given the additive benefits observed with probiotics and the preservation of muscle mass, future studies should investigate combination therapies, such as LOLA with GLP‐1 receptor agonists, to determine if synergistic effects can provide a comprehensive management strategy for the metabolic, fibrotic and sarcopenic components of the disease.

## 5 CONCLUSION

In conclusion, this systematic review synthesizes a robust body of experimental and clinical evidence supporting the therapeutic potential of LOLA in MASLD. Preclinical models elucidate that LOLA exerts pleiotropic hepatoprotective effects not only through ammonia scavenging but also via the restoration of mitochondrial bioenergetics, activation of the LKB1‐AMPK signalling pathway and modulation of the gut‐liver‐muscle axis. Clinically, the analysis of nine studies involving over 1600 patients demonstrates that LOLA intervention consistently normalizes liver enzymes and blood ammonia levels, while improving surrogate markers of steatosis and fibrosis, intrahepatic hemodynamics and patient‐reported outcomes such as fatigue and cognitive function. These findings suggest that LOLA is a safe and mechanistically sound candidate for repurposing as an adjunct therapy to target the metabolic and fibrotic drivers of MASLD. However, to definitively establish its role in clinical practice guidelines, future research utilizing rigorous randomized controlled trials with the standardized MASLD diagnostic criteria and histological endpoints is essential.

## AUTHOR CONTRIBUTIONS

A.I. had the idea of the manuscript. A.I. and V.C. independently applied the search strategy and performed the study selection. A.I. and V.C. performed risk of bias assessment. A.I., V.C. and S.L.P. performed the data extraction. A.I., V.C. and S.L.P. drafted the manuscript. D.L.D. contributed to the writing of the manuscript. A.I. and D.L.D. made substantial contributions to the conception and critically revised the manuscript for important intellectual content. All authors revised the final manuscript and approved the final version.

## FUNDING INFORMATION

The authors did not receive any financial support for the research, authorship and/or publication of this article.

## CONFLICT OF INTEREST STATEMENT

The authors declare that the research was conducted in the absence of any commercial or financial relationships that could be construed as a potential conflict of interest.

## Supporting information


Table S1.

## Data Availability

Data available in Tables 1 and 2.
