# Supplementary material for: Can ammonia scavenging treat MASLD? Evaluating the evidence for L‐ornithine L‐aspartate—A systematic review
Source: Eur J Clin Invest. 2026 Feb 7;56(2):e70185. doi: 10.1111/eci.70185 (PMC12882027; doi:10.1111/eci.70185)
Supplement: Supplementary file 1 — Table S1. [file ECI-56-e70185-s001.docx]

**Supplementary Table S1.** Quality Assessment of Included Experimental Studies (SYRCLE's Risk of Bias Tool)

| **Study (Year)** | **Sequence Generation** | **Baseline Characteristics** | **Allocation Concealment** | **Random Housing** | **Blinding (Caregivers)** | **Random Outcome Assessment** | **Blinding (Outcome Assessor)** | **Incomplete Outcome Data** | **Selective Reporting** | **Other Sources of Bias** |
| --- | --- | --- | --- | --- | --- | --- | --- | --- | --- | --- |
| **Prikhodko et al. (2020)** | 🟢 **Low** | 🟢 **Low** | 🟡 **Unclear** | 🟢 **Low** | 🟡 **Unclear** | 🟡 **Unclear** | 🟡 **Unclear** | 🟢 **Low** | 🟢 **Low** | 🟢 **Low** |
| **Oleshchuk et al. (2021)** | 🟢 **Low** | 🟢 **Low** | 🟡 **Unclear** | 🟡 **Unclear** | 🟡 **Unclear** | 🟡 **Unclear** | 🟡 **Unclear** | 🟢 **Low** | 🟢 **Low** | 🟢 **Low** |
| **Rao et al. (2021)** | 🟢 **Low** | 🟢 **Low** | 🟡 **Unclear** | 🟢 **Low** | 🟡 **Unclear** | 🟡 **Unclear** | 🟢 **Low** | 🟢 **Low** | 🟢 **Low** | 🟢 **Low** |
| **de Freitas et al. (2022)** | 🟢 **Low** | 🟢 **Low** | 🟡 **Unclear** | 🟢 **Low** | 🟡 **Unclear** | 🟡 **Unclear** | 🟢 **Low** | 🟢 **Low** | 🟢 **Low** | 🟢 **Low** |
| **Pichon et al. (2022)** | 🟢 **Low** | 🟢 **Low** | 🟡 **Unclear** | 🟢 **Low** | 🟡 **Unclear** | 🟡 **Unclear** | 🟢 **Low** | 🟢 **Low** | 🟢 **Low** | 🟢 **Low** |
| **Canbay et al. (2024) *** | N/A | 🟢 **Low** | N/A | N/A | 🟡 **Unclear** | N/A | 🟡 **Unclear** | 🟢 **Low** | 🟢 **Low** | 🟢 **Low** |
| **Guo et al. (2024)** | 🟢 **Low** | 🟢 **Low** | 🟡 **Unclear** | 🟢 **Low** | 🟡 **Unclear** | 🟡 **Unclear** | 🟡 **Unclear** | 🟢 **Low** | 🟢 **Low** | 🟢 **Low** |
| **Lange et al. (2024)** | 🟢 **Low** | 🟢 **Low** | 🟡 **Unclear** | 🟢 **Low** | 🟡 **Unclear** | 🟡 **Unclear** | 🟡 **Unclear** | 🟢 **Low** | 🟢 **Low** | 🟢 **Low** |
| **Longo et al. (2024)** | 🟢 **Low** | 🟢 **Low** | 🟡 **Unclear** | 🟢 **Low** | 🟡 **Unclear** | 🟡 **Unclear** | 🟢 **Low** | 🟢 **Low** | 🟢 **Low** | 🟢 **Low** |
| **Su et al. (2024)** | 🟢 **Low** | 🟢 **Low** | 🟡 **Unclear** | 🟢 **Low** | 🟡 **Unclear** | 🟡 **Unclear** | 🟡 **Unclear** | 🟢 **Low** | 🟢 **Low** | 🟢 **Low** |
| **Low**: Low risk of bias; **High**: High risk of bias; **UNCLEAR**: Unclear risk of bias (insufficient details reported); **N/A**: Not applicable.  **Note**: Canbay et al. (2024) is an in vitro study; therefore, domains related to animal housing and allocation concealment are marked as N/A. | | | | | | | | | | |

**Supplementary Table S2.** Quality Assessment of Randomized Controlled Trials (Cochrane Risk of Bias Tool)

| **Study (Year)** | **Random Sequence Generation** | **Allocation Concealment** | **Blinding of Participants & Personnel** | **Blinding of Outcome Assessment** | **Incomplete Outcome Data** | **Selective Reporting** | **Other Bias** | **Overall Risk of Bias** |
| --- | --- | --- | --- | --- | --- | --- | --- | --- |
| **Tian et al. (2013)** | 🟢 **Low** | 🟡 **Unclear** | 🟡 **Unclear** | 🟢 **Low** | 🟢 **Low** | 🟢 **Low** | 🟢 **Low** | **Low** |
| **Zhuravlyova et al. (2015)** | 🟢 **Low** | 🟡 **Unclear** | 🔴 **High** | 🟡 **Unclear** | 🟢 **Low** | 🟢 **Low** | 🟢 **Low** | **Moderate** |
| **Ilchenko et al. (2016)** | 🟢 **Low** | 🟡 **Unclear** | 🔴 **High** | 🟡 **Unclear** | 🟢 **Low** | 🟢 **Low** | 🟢 **Low** | **Moderate** |
| **Low:** Low risk of bias; **High:** High risk of bias; **Unclear:** Insufficient information to permit judgment. | | | | | | | | |

**Supplementary Table S3.** Quality Assessment of Observational and Non-Randomized Studies (Newcastle-Ottawa Scale)

| **First Author (Year)** | **Study Design** | **Selection (Max 4 stars)** | **Comparability (Max 2 stars)** | **Outcome (Max 3 stars)** | **Total Score (Max 9)** | **Quality Level** |
| --- | --- | --- | --- | --- | --- | --- |
| **Grüngreiff et al. (2001)** | Prospective Cohort | ★★★ | ★ | ★★ | **6** | Good |
| **Ageeva et al. (2017)** | Comparative Study | ★★★ | ★ | ★★ | **6** | Good |
| **Sas (2018)** | Observational | ★★ | - | ★★ | **4** | Fair |
| **Kizova et al. (2019)** | Observational | ★★ | - | ★★ | **4** | Fair |
| **Ermolova et al. (2020)** | Comparative Study | ★★★ | ★ | ★★ | **6** | Good |
| **Garanina (2021)** | Case-Control/Cohort | ★★★ | ★ | ★★ | **6** | Good |
